# HUMAN FACTORS ISSUES IN THE DESIGN OF VIRTUAL REALITY TO SUPPORT OLDER ADULTS’ SOCIAL AND COGNITIVE ENGAGEMENT

**DOI:** 10.1093/geroni/igad104.3401

**Published:** 2023-12-21

**Authors:** Veronica Falcon Borbolla, Walter Boot, Andrew Dilanchian, Ana Sophia Garces Torres, Mario Hernandez, Bill Tong Xu, Armin Mostafavi, Benjamin Kim

**Affiliations:** Florida State University, Tallahassee, Florida, United States; Florida State University, Tallahassee, Florida, United States; Florida State University, Tallahassee, Florida, United States; Weill Cornell Medicine, New York City, New York, United States; Weill Cornell Medicine, New York City, New York, United States; Cornell University, Ithaca, New York, United States; Cornell University, Ithaca, New York, United States; Cornell University, Ithaca, New York, United States

## Abstract

Emerging technologies such as virtual reality (VR) hold tremendous potential to improve the lives of older adults, including the opportunity to support social and cognitive engagement. However, this potential will only be realized if VR technologies and applications (apps) are well-designed, considering the needs, preferences, and abilities of older people. To explore and understand design barriers ahead of a randomized controlled trial testing the efficacy of a psychosocial VR intervention for older adults, a team of eight evaluators who underwent standardized and comprehensive training on how to perform a heuristic analysis assessed the design of 28 commercial VR apps. Apps were chosen for their potential to support social interactions, enhance cultural opportunities, facilitate new learning, or support leisure activities. Each app was evaluated based on the Nielsen Norman Group’s ten usability heuristics. For different VR components (hardware, app menus, VR environment), this analysis highlighted specific heuristic violations, examined the general human factors of these apps, and identified positive design elements while suggesting solutions for observed heuristic violations. The heuristic analysis revealed significant usability challenges, especially in areas like “System Status Visibility,” “User Control and Freedom,” and “Help and Documentation.” The primary system components exhibiting these heuristic violations were the “User Interface” and “Virtual Environment.” It was concluded that current virtual reality systems do not adequately address human factors issues, particularly considering the needs and abilities of older adult users. Based on this extensive evaluation, training and design solutions will be presented and VR guidelines for older adults will be introduced.

